# Neoadjuvant Therapies for Prostate Cancer–Current Paradigms and Future Directions

**DOI:** 10.3390/cancers18010065

**Published:** 2025-12-24

**Authors:** Kieran Sandhu, Abdullah Al-Khanaty, David Hennes, David Chen, Eoin Dinneen, Carlos Delgado, Nathan Lawrentschuk, Renu S. Eapen, Declan G. Murphy, Marlon Perera

**Affiliations:** 1 Division of Cancer Surgery, Peter MacCallum Cancer Centre, Parkville, VIC 3000, Australia; 2 University of Cambridge, Cambridge CB2 3PT, UK; 3 Department of Surgery, University of Melbourne, Austin Health, Melbourne, VIC 3010, Australia; 4 Hudson Institute of Medical Research, Monash University, Clayton, VIC 3800, Australia; 5 Department of Surgery, University of Melbourne, Royal Melbourne Hospital, Melbourne, VIC 3050, Australia; 6 EJ Whitten Prostate Cancer Research Centre, Epworth Healthcare, Melbourne, VIC 3002, Australia; 7 Sir Peter MacCallum Department of Oncology, University of Melbourne, Melbourne, VIC 3010, Australia

**Keywords:** neoadjuvant therapy, prostate cancer, ARPIs, ADT, radioligand therapy

## Abstract

High-risk and locally advanced prostate cancer represents 20–25% of new diagnoses of prostate cancer and is associated with high rates of recurrence, morbidity, and mortality. The neoadjuvant window provides a unique opportunity for systemic control prior to definitive therapy with radical prostatectomy or radiotherapy (RT). Early trials with first-generation androgen deprivation therapy (ADT) achieved pathological downstaging but no survival benefit. In the 2000s, the advent of chemohormonal regimes using docetaxel provided excitement but mixed results tempered expectations and is now not recommended prior to surgery. Second-generation androgen receptor pathway inhibitors (ARPIs) combined with ADT have demonstrated significant survival benefit in metastatic prostate cancer and are currently being evaluated in large phase III trials in the neoadjuvant setting. RT remains an alternative curative modality, and recent data highlights similar issues to surgery in eradicating micrometastatic disease despite excellent local control. This has driven parallel efforts to evaluate intensified systemic therapy in the pre-RT/neoadjuvant settings. In addition to the excitement surrounding ARPIs, radioligand therapy, such as [^177^Lu]Lu-PSMA-617 has shown promise in the neoadjuvant setting and continues to be investigated. Future research aims to incorporate genomic and molecular factors to enable personalised neoadjuvant therapies by identifying damage immunologically responsive subtypes that may derive greater benefit from immune-directed therapies in the peri-operative setting. This narrative review synthesises current evidence for neoadjuvant therapies in high-risk prostate cancer and future directions.

## Simple Summary

High-risk prostate cancer has a high chance of return despite surgery or radiotherapy. Treatment prior to definitive therapy (neoadjuvant therapy) may help control cancer earlier and reduce spread. Early hormonal therapy improved tumour features but not survival. Second-generation anti-hormonals, radioligand therapy, and immunotherapy are now being explored in the neoadjuvant setting. This review summarises current evidence and ongoing trials exploring how neoadjuvant treatments may improve outcomes for men with aggressive prostate cancer.

## 1. Introduction

Prostate cancer (PCa)_ is the second most commonly diagnosed malignancy in men worldwide, with over 1.5 million new cases diagnosed annually and approximately 397,000 prostate cancer-related deaths globally [1]. High-risk, locally advanced PCa accounts for approximately 20–25% of new diagnoses in Western countries and remains a major cause of PCa-specific mortality and morbidity [2,3]. The incidence of high-risk and locally advanced disease varies by region, reflecting differences in screening practices and access to healthcare. Despite advances in multimodal therapy, outcomes in this group remain heterogeneous, reflecting the biological complexity and variable natural history of aggressive disease.

Radical prostatectomy (RP) remains integral to curative-intent management, offering the potential for local control and pathological staging. However, disease recurrence following surgery remains a persistent challenge, with up to 50% of men experiencing biochemical recurrence (BCR) within a decade of surgery [4,5]. This underscores the need for improved strategies targeting both micrometastatic and locally residual disease at an earlier stage. Radiotherapy (RT) represents another established curative modality for high-risk PCa, typically delivered alongside androgen deprivation therapy (ADT). Despite excellent local control with modern RT regimes, a proportion of patients will still develop biochemical recurrence (BCR), highlighting similar issues as to surgery [6]. These parallels further support the need for peri-radiation systemic treatment intensification; mirroring efforts made in the surgical neoadjuvant setting.

The peri-operative window provides a unique opportunity for treatment intensification with systemic therapy. Early trials with neoadjuvant ADT prior to RP improved pathological findings, including reductions in tumour volume, pathological downstaging, and margin negativity, yet these did not translate to overall survival (OS) benefits [7]. Subsequent studies incorporating chemotherapy with docetaxel provided mixed, and largely discouraging resulted and is not recommended in the neoadjuvant setting prior to RP [8]. Comparatively, for RT neoadjuvant provided significant benefit and remains integral to therapy [9,10,11,12].

Recently, the emergence of androgen receptor pathway inhibitors (ARPI) therapy has redefined systemic therapy in advanced and metastatic PCa, with early-phase trials demonstrating encouraging rates of pathological response and molecular downstaging [13]. In parallel, multiple phase II and III trials are currently underway evaluating the efficacy of ARPI-based systemic intensification before, or during, RT, reflecting a broader shift towards combing AR suppression with local therapy to provide long-term systemic disease control [14,15].

The evolution of molecular imaging and theranostics has opened new frontiers in peri-operative therapy. Prostate-specific membrane antigen (PSMA) targeted radioligand therapy (RLT), such as [^177^Lu]Lu-PSMA-617, has demonstrated efficacy in advanced disease and is now being explored in the neoadjuvant and adjuvant settings to eradicate micrometastatic disease [16].

Current international guidelines emphasise the need for multi-modal approaches for high-risk disease [17,18].

This narrative review intended to synthesise contemporary evidence on neoadjuvant systemic therapies in high-risk PCa. Studies were selected based on clinical relevance, trial phase, and impact on current or emerging treatment paradigms, with an emphasis on randomised trials, large prospective studies, and translational analyses. Where applicable, trials in the metastatic setting were included to rationalise treatment intensification in earlier disease settings. Furthermore, we explore the evolving role of next-generation ARPIs, immunotherapy, and PSMA-targeted theranostics in the contemporary management paradigm.

## 2. Biological Rationale for Neoadjuvant Therapy

**Figure 1:**
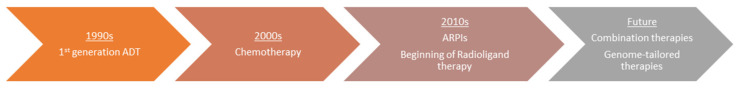
Timeline of neoadjuvant therapies in prostate cancer.

In locally advanced PCa, cellular dissemination may have already occurred, remaining dormant in distant tissue and reactivating years after local therapy [19]. Peri-operative systemic therapy may enable elimination of these clones and suppression of any inflammatory response that promotes metastatic outgrowth [20]. Neoadjuvant trials use a range of pathological and clinical endpoints to evaluate biological activity and long-term benefit. Pathological complete response (pCR) refers to the absence of residual viable tumour in the prostate and lymph nodes, while minimal residual disease (MRD) denotes typically ≤5% residual tumour volume after therapy. Both pCR and MRD serve as surrogate markers of intraprostatic tumour eradication and reflect depth of response to neoadjuvant treatment. Validated clinical endpoints include BCR, defined as a post-operative PSA rise suggesting persistent or recurrent disease, metastasis-free survival (MFS) capturing distant disease control, and overall survival (OS) [21]. These outcomes provide insight into whether neoadjuvant-mediated tumour reduction achieves a meaningful long-term benefit for patients. Therefore, neoadjuvant therapy provides an opportunity to assess for clonal selection and resistance evolution through both pre- and post-treatments specimens. A summary of recently reported and ongoing trials is provided in Table 1. Additionally, Figure 1 summarises the evolving landscape of neoadjuvant systemic therapies in high-risk PCa, highlighting the transition from cytoreductive strategies to multimodal intensification.

## 3. First-Generation ADT

Neoadjuvant ADT in high-risk PCa dates to the early 1990s where it was given to reduce tumour volume prior to RP. Multiple trials evaluated luteinising hormone-releasing hormone (LHRH) agonists or antagonists, with or without first-generation anti-androgens such as bicalutamide, administered over three to six months prior to surgery [33,34,35,36]. Neoadjuvant ADT demonstrated significant pathological improvements including reduction in prostate volume, pathological tumour stage, seminal vesicle invasion, and the incidence of positive surgical margins [33,34,35,37]. Building on these initial trials, meta-analyses confirmed these benefits showing up to a 50% reducing in margin positivity rates, and improved pathological downstaging across cohorts [38,39].

Despite these promising pathological gains, no study was able to demonstrate a meaningful improvement in long-term outcomes including OS, MFS, or biochemical recurrence-free survival (BCRFS). Kotz and colleagues—part of the Canadian Urologic Oncology Group—randomised patients to either RP alone or 12 weeks of cyproterone acetate or flutamide with goserelin before RP [33]. Despite the neoadjuvant arm exhibiting tumour-downstaging, they found no differences in five-year progression-free survival (PFS) between groups [33]. Further studies with extended-treatment comparing three verses eight months of ADT had the same findings [29].

Beyond surgery, neoadjuvant ADT has been long evaluated in combination with RT. Multiple landmark, RT-based trials—including RTOG 86–10 and TROG 96.01—have demonstrated that 2–6 months of ADT prior to, or during, RT significantly reduces rates of local recurrence, distant metastases, and prostate cancer-specific mortality and BCRFS [9,10,11,12]. In these landmark trials, short-course neoadjuvant ADT provided significant survival benefit, helping to establish combination ADT-RT as a foundational therapeutic regime in patients with high-risk PCa. Furthermore, randomised trials including EORTC 22961, RTOG 92-02, and DART 01/05 have established that long-term ADT (18–36 months) combined with RT significantly improves OS and MFS compared with short-term ADT in patients with high-risk and locally advanced PCa [11,40,41].

### 3.1. Biological and Methodological Explanations

Traditional LHRH-based ADT achieves castrate testosterone levels but may not entirely suppress adrenal or intra-tumoural androgen synthesis [42]. This may select for subclones that can persist in low-androgen environments via AR amplification or alternative means of steroidogenesis [43]. Early trials in ADT included low- and intermediate-risk patients, which may dilute the biological benefit in aggressive disease where systemic control is most imperative. The benefit of short-course ADT is its cytoreductive effect, rather than providing durable eradication of disseminated tumour cells which likely explains the absence of MFS or OS benefit. In terms of RT, ADT not only provides a cytoreductive effect, but improves RT dosimetry and suppresses AR-mediated DNA repair, which thereby enhances tumoural radiosensitivity [44,45,46]. These findings reinforce that while ADT alone before RP failed to improve survival, its integration with RT has proven oncologically meaningful and beneficial.

### 3.2. Lessons and Contemporary Insights

Early ADT established an important proof-of-concept that neoadjuvant therapy could safely, and effectively, alter biological tumour features. However, it also highlighted that cytoreduction of the primary tumour and local pathological improvement does not necessarily equate to survival benefit, which may limit the prognostic value of pathological downstaging in these patients. Despite these findings in the surgical context, modern RT trials have confirmed that androgen suppression biologically primes a tumour for radiation-induced DNA damage, while suppression of AR signalling heightens the tumour’s sensitivity to RT by blocking non-homologous DNA end-joining repair [44,45,46]. Nonetheless, recent insights into intratumoural androgen synthesis, androgen receptor mutations, and mutations DNA damage response (DDR) pathways provides a biological rationale for intensification of hormonal blockade with other therapies [42,47,48]. These insights are reflected by many contemporary guidelines—including the National Comprehensive Cancer Network (NCCN), European Association of Urology (EAU), and the American Urological Association (AUA) [4,49,50].

## 4. Chemotherapy

Much of the rationale for neoadjuvant systemic intensification in high-risk localised PCa is derived from pivotal trials in the metastatic setting, which established survival benefits and effective control of systemic disease. Interest in chemotherapy for prostate cancer was reinvigorated in the early 2000s with the introduction of docetaxel. The pivotal TAX 327 and CHAARTED trials demonstrated a clear overall survival benefit in patients with metastatic or advanced disease, marking the first significant improvement in systemic outcomes for this cohort [51,52]. These landmark findings prompted a paradigm shift and sparked growing interest in exploring the role of docetaxel earlier in the disease continuum, including its potential integration into the peri-operative and neoadjuvant settings for high-risk, localised prostate cancer [51,52]. The rationale for neoadjuvant chemotherapy was that cytotoxic chemotherapy may be an adjunct to androgen suppression, targeting proliferating androgen-independent subclones prior to RP. Taxanes, including docetaxel and cabazitaxel, act synergistically with ADT by inhibiting microtubule depolymerisation and directly blocking AR nuclear translocation, thus enhancing AR signalling blockage [53]. Thus, the combination of ADT and docetxael, in theory, offers a two-pronged approach—suppression of androgen-mediated proliferation and cytotoxicity for resistant subclones.

Beyond the surgical setting, taxane chemotherapy has been integrated into the adjuvant RT setting for high-risk disease. The phase III RTOG 0521 trial demonstrated that adding docetaxel to long-term ADT and dose-escalated RT significantly improved outcomes, with higher 4-year OS in patients treated with ADT + RT + Chemotherapy compared to ADT + RT alone (93%, 95% CI: 90–96% vs. 89%, 95% CI: 84–92%, respectively) [54]. Despite these findings, use is only suggested in contemporary guidelines as an escalation strategy in appropriately selected, fit men with high risk non-metastatic PCa [4,49,50]. Nonetheless, ongoing and trials awaiting publication will further refine the role of systemic intensification in RT-based treatment paradigms. Notably, the PEACE-2 trial is evaluating the integration of carbazitaxel with ADT and RT in patients with high-risk PCa [27]. The results of this study are anticipated to clarify whether next-generation taxanes can improve outcomes beyond established chemoradiotherapy approaches.

### 4.1. Evidence from Trials

Initial phase I/II trials by Febbo et al., and Chi et al., demonstrated the feasibility and safety of neoadjuvant chemotherapy in providing marked cytoreduction and reducing positive surgical margin rates, without compromising surgical outcomes [8,55]. However, these studies were limited by small sample sizes and short follow-up time. The landmark CALGB 90203 (Alliance) trial randomised 788 men to either RP alone or six cycles of Docetaxel and ADT prior to RP [26]. Although the primary outcome, BCRFS, was not met, the chemohormonal arm demonstrated significant improvements in MFS (HR 0.70, 95% CI 0.51–0.95) and OS (HR 0.61, 95% CI 0.40–0.94) [26]. Importantly, there were no differences in surgical complication rates or recovery between groups, supporting the safety of chemohormonal therapy in the neoadjuvant setting [26]. Despite the evidence supporting the long-term benefits of systemic intensification before definitive surgical management, the increased toxicity observed in the chemotherapy group limited the routine use of chemohormonal therapy in localised high-risk PCa. In the RT setting, phase I/II studies have demonstrated feasibility and low toxicity profiles of docetaxel administered concurrently with RT [56,57,58]. This data suggests that taxane-based radiosensitisation may enhance the cytotoxic effect of RT, although robust randomised evidence for pre-RT chemotherapy is lacking.

Several contemporary studies have built on Alliance, exploring taxane-based approaches with next-generation hormonal therapy. The ACDC-RP phase II study enrolled 70 patients to receive abiraterone acetate and LNRH with or without cabazitaxel prior to RP [25]. Across both arms, 44% of men achieved pathological complete response (pCR) or minimal residual disease (MRD) with excellent tolerability [25]. Other small series have assessed the combination of chemotherapy and ARPIs demonstrating excellent volumetric tumour reduction but achieving low pathological complete pCR rates [59]. These data suggests that chemohormonal therapy provides excellent cytoreduction; however, complete pathological eradication remains rare, likely due to the development of therapeutic resistance mechanisms by subclones.

Evidence from meta-analyses support the safety of docetaxel but have demonstrated only modest short-term pathological benefit. A pooled analysis of over 1000 patients showed that chemohormonal therapy reduced the risk of positive surgical margins by approximately 65%, risk of seminal vesicle invasion by 22%, and increased the probability of pathological downstaging by 64% compared to RP alone [60]. However, across these analyses the survival improvements were inconsistent, reflecting methodological heterogeneity.

### 4.2. Current Position of Chemotherapy

The dual-pronged chemohormonal approach has demonstrated proof of concept that systemic intensification can achieve benefit in selected high-risk patients, but widespread adoption in the neoadjuvant setting is not recommended. Chemohormonal therapy may provide benefit in select very high-risk subgroups, such as those patients with *TP53* or *PTEN* loss, which confers androgen-independent growth [61].

## 5. ARPIs

Second-generation ARPIs—abiraterone acetate, enzalutamide, apalutamide, and darolutamide—have fundamentally reshaped the therapeutic landscape of metastatic prostate cancer, providing substantial overall survival benefits across hormone-sensitive and castration-resistant disease states [62,63,64,65]. By targeting distinct nodes within the androgen receptor axis, these agents achieve a more profound suppression of androgen receptor signalling compared to first-generation antiandrogens. Their success in the advanced setting has naturally catalysed growing interest in their application within the neoadjuvant and peri-operative context, where early intervention may enhance pathological response, reduce tumour burden, and potentially mitigate the risk of micrometastatic progression following radical prostatectomy.

### 5.1. Evidence from Phase II Trials

Several phase II trials have evaluated ARPI-based strategies in combination with LHRH therapies, with durations ranging from 3 to 6 months. In their randomised study, McKay et al. compared enzalutamide + LHRH versus enzalutamide + abiraterone/prednisolone + LHRH in 75 men with intermediate- or high-risk PCa [13]. Both groups demonstrated similar rates of PSA suppression and high rates of pCR, but differences were not significant between the two arms. Both arms demonstrated a similar reduction in rates of T3 disease and positive margins [13]. In their randomised, placebo-controlled trial (ARNEO), Devos et al., assigned 89 men with high-risk localised PCa to Degarelix plus or minus Apalutamide [22]. Compared to Degarelix alone, the combination arm achieved significantly higher rates of MRD (38% vs. 91%, respectively) [22]. Patients with *PTEN* loss at diagnostic biopsy displayed significantly less MRD than those without *PTEN* loss, highlighting the inherent androgen-insensitive growth in these patients [22].

Parallel to surgical neoadjuvant ARPI studies, ARPI intensification therapy has also become a major focus in the RT setting. The ENZARAD phase III trial (ANZUP 1303/NCT0244644) randomised 802 men with high-risk localised, or locally advanced, PCa to enzalutamide + ADT + RT versus standard non-steroidal anti-androgen therapy + ADT + RT, with interim analyses confirming acceptable genitourinary toxicity with select benefit on MFS in patients with N1 disease and planned pelvic RT, but not in those with very-high risk disease—cN1 or Grade Group 4–5 disease with cT2b-4 or PSA > 20 ng/mL [14]. Similarly, the ATLAS trial (NCT0253516) is evaluating the role of apalutamide + ADT + with results pending [15]. The STAMPEDE platform has provided high level evidence—adding abiraterone (Arm G) or abiraterone + enzalutamide to ADT + RT significantly improves MFS in men with non-metastatic high-risk disease, establishing ARPI-based systemic intensification around RT as an emerging standard [66].

### 5.2. Translational Insights

Tissue analyses in patients receiving neoadjuvant ARPIs have revealed important molecular mechanisms. Firstly, *PTEN*-deficiency contributes to higher MRD [22]. Secondly, residual tumour following ARPI treatment may exhibit significantly higher glucocorticoid receptor protein expression than baseline, which correlates with shorter BCRFS [67]. Thirdly, ARPI therapy may stimulate an immune response with both complement and macrophage activation, highlighting the potential for synergistic approaches combining AR blockage and immune checkpoint inhibition [68]. Although final data is pending, PROTEUS is a Phase III trial that has randomised approximately 2000 men with high-risk, or locally advanced PCa to Apalutamide + ADT versus placebo + ADT administered pre- and post-RP [23].

## 6. Radioligand Therapy

RLT leverages selective expression of PSMA, which is upregulated in PCa epithelial cells and endothelial cells of neovasculature [69]. PSMA is an ideal target as its expression increases proportionally with aggressive disease [70]. A popular approach is the combination of PSMA with the β-emitting radionuclide lutetium-177 ([^177^Lu]Lu-PSMA-617), facilitating cancer-specific cytotoxicity [71,72]. In the metastatic setting, [^177^Lu]Lu-PSMA-617 has been validated in the large TheraP and VISION trials, demonstrating significant survival benefit for patients with metastatic castrate-resistant PCa [71,73]. Given these advances, there is significant interest in exploring the role of RLT in the neoadjuvant setting in the context of high- and very-high-risk localised PCa [74]. Beyond direct tumour cytotoxicity, RLT exerts immunomodulatory effects, including induction of DNA damage, release of neoantigens, and activation of innate and adapative immune pathways [75].

The LuTectomy study by Eapen et al. enrolled 20 patients with grade group 3–5 PCa, a PSA > 20 ng/mL, cT2+, N1 disease, and high PSMA uptake (SUV_max_ > 20) [16]. Participants received a single 5 GBq dose of [^177^Lu]Lu-PSMA-617 six weeks prior to RP. Surgery was uncomplicated in all patients, and 45% of patients achieved a >50% reduction in their PSA pre-operatively, with few mild adverse events reported. Similarly, Golan et al. conducted a single-arm feasibility study giving men two to three cycles of [^177^Lu]Lu-PSMA-617 six weeks prior to RP achieving a median PSA decline of 34% prior to RP following three doses, with excellent tolerability [30]. Neoadjuvant RLT remodels the tumour microenvironment, triggering infiltration of CD8^+^ T cells, activation of type I interferon signalling, and upregulation of immune checkpoint molecules (including PD-L1), which provides a rationale for combination therapy with immunotherapy [74].

### Future Directions and Perspectives

β-emitters such as [^177^Lu]Lu-PSMA-617 delivers radiation with moderate tissue penetration of approximately 1–2 mm, while α-emitting isotopes, including actinium-225 and thorium-227, release particles with shorter particles lengths (<100 μm) [76]. This produces localised DNA breaks, which could be of benefit in small, residual tumour foci. Early case reports and pilot series with [^225^Ac]Ac-PSMA-617 have shown benefit in PSA reductions and durable control in the metastatic castrate resistant PCa setting [76]. Theoretically, these agents may achieve deeper tumour penetration than β-emitters, though careful monitoring and dosimetry and important given the potential for side effects.

Additionally, sequential or combination strategies are now under investigation including [^177^Lu]Lu-PSMA-617 + ARPI, [^177^Lu]Lu-PSMA-617 + PARP inhibition, and [^177^Lu]Lu-PSMA-617 + PD-L1 inhibition [77,78]. Across these studies, neoadjuvant RLT has demonstrated excellent tolerability without impairing surgical efficacy. Most reported side effects are grade I–II in nature and are transient. Critically, RLT has not impaired the ability to perform a nerve-sparing procedure, or any impact on continence recovery [16]. Although there is certainly considerable promise in neoadjuvant RLT, most work to-date has been exploratory or feasibility studies, and future success and dissemination will require work to identify optimal dosing and timing, and appropriate patient selection.

## 7. Genomic and Immunologic Strategies

Advances in next-generation sequencing have enabled identification of distinct genomic and immune subtypes with therapeutic targetability, commonly arising due to alterations in DDR genes and immune checkpoint pathways. These insights have paved the way for targeted neoadjuvant therapy.

### 7.1. DDR-Directed Therapy and PARP Inhibition

Defects in homologous recombination repair (HRR) genes—BRCA 1/2, ATM, CHEK2, and PLAB 2—are common in high-risk PCa [48]. Loss of HRR function sensitises these tumours to PARP inhibition, whereby blocking of PARP prevents repair of single-stranded DNA breaks, subsequent double-strand DNA damage and cell death in DDR-deficient cells [79,80,81]. In advanced disease, particularly BRCA 1/2 mutational subsets, PARP inhibitors have shown significant improvement in PFS [82]. The NePtune trial (NCT05498272) is the first trial to examine the role of neoadjuvant olaparib in high-risk localised PCa [28]. Men with BRCA 1/2 mutations and high-risk or locally advanced PCa will receive six months of Olaparib prior to RP, with the primary endpoint being pCR and MRD.

### 7.2. Immune-Targeted Approaches

Many cases of PCa are sparsely populated by immune infiltrates [83,84]. Thus, these tumours are commonly described as immunologically “cold”, which has historically limited the efficacy of immune checkpoint inhibition in unselected cases, necessitating combination strategies to enhance tumour immunogenecity [84,85]. However, subsets of patients, including those with DDR deficiencies, mismatch repair deficiency, CDK12 alterations, or high tumour mutational burden, exhibit heighted neoantigen expression and thus immune responsiveness [86,87]. This biological heterogeneity provides a rationale for biomarker-driven immune-targeted strategies in high-risk PCa.

A promising target is *B7-H3*, which is an immune checkpoint molecule overexpressed in PCa and the neovasculature, associated with more aggressive disease and poor prognosis [88]. In their phase II trial, Shenderov et al. gave 32 men with high-risk, localised PCa Enoblituzumab demonstrating excellent tolerability, and 66% of patients achieved an undetectable PSA one-year post-RP [32]. Of those that responded, there was a significantly higher pre-treatment CD8^+^ T cell infiltrate supporting the concept that baseline immune priming may predict response to immune-based therapies [32].

PSMA-targeted therapy, including [^177^Lu]Lu-PSMA-617 may enhance immune priming by induction of DNA damage and release of neoantigen, with subsequent activation of innate and adaptive immune pathways and upregulation of immune checkpoint signalling, providing rationale for RLT and immunotherapy dual therapy [89,90]. These immunomoedulatory effects have strengthened interest in rational combination strategies integrating RLT with immune checkpoint blockage. The PRINCE trial (NCT05150236) is investigating the feasibility of [^177^Lu]Lu-PSMA-617 + Pembrolizumab in metastatic castrate-resistant PCa [77].

## 8. Future Directions and Perspective

Future progress in the neoadjuvant setting will depend on the integration of systemic intensification with precise patient selection. Biomarker-driven approaches incorporating patient-specific genomic alterations (for example, DDR status, *PTEN* loss), advanced imaging, and circulating tumour markers are likely to refine treatment allocation and identify patients most likely to benefit from neoadjuvant treatment escalation. The use of composite endpoints beyond pathological response will be crucial to comprehensive assessment of emerging strategies. Ultimately, the goal of neoadjuvant therapy will be not only tumour downstaging, but durable suppression of systemic disease and improved long-term survival.

## 9. Conclusions

Neoadjuvant therapy in PCa is rapidly redefining the management of high-risk and locally advanced disease in both the surgical and RT settings. Early clinical trials have demonstrated that ARPI-based regimens achieve profound intraprostatic androgen suppression and consistent pathological downstaging, supporting their feasibility and biological efficacy. Concurrently, the integration of emerging modalities such as PSMA-targeted radioligand therapy (RLT) and immunotherapy is broadening the therapeutic horizon, offering novel avenues for multimodal synergy.

However, while these strategies yield compelling surrogate endpoints, durable survival benefit remains unproven, and long-term oncologic outcomes will determine their true clinical value, though studies are currently being conducted that will report on this. Current international guidelines do not routinely recommend neoadjuvant systemic therapy prior to RP outside clinical trials, while endorsing ADT in combination with RT as standard of care for high-risk and locally advanced PCa. The next phase of progress, and success, will hinge on precision-based approaches that integrate molecular profiling, imaging biomarkers, and genomic risk stratification to tailor neoadjuvant treatment intensity and sequence.

Ultimately, the goal of neoadjuvant therapy in PCa should extend beyond local tumour downstaging—it should aim to fundamentally alter the systemic course of the disease, bridging surgical and systemic oncology to achieve deeper, more durable remission in patients with biologically aggressive prostate cancer. The success of neoadjuvant therapy will depend not only on tumour downstaging but also on its ability to alter the systemic trajectory of disease through biologically informed, patient-specific treatment intensification.

## Figures and Tables

**Table 1:** Pivotal completed and ongoing neoadjuvant trials in high-risk and locally advanced prostate cancer.

Authors/Trial	Phase/Design	Eligibility/Population	Therapy	Key Findings
ARPI ± ADT				
ARNEO [22]	Randomised Phase II	High-risk localised PCa (Gleason score ≥ 8, PSA > 20 or cT3)	Degarelix ± Apalutamide vs. placebo for 3 months prior to RP	MRD achieved in 38% vs. 9.1% Benefit for *PTEN*-intact
PROTEUS [23]	Double-blind, placebo-controlled Phase III	High-risk/locally advanced	Apalutamide + ADT vs. Placebo + ADT for 6 months pre- and post-RP	Ongoing with ~1500 patients Results expected 2026
McKay et al. [13]	Randomised Phase II	Intermediate- or high-risk localised PCa (*n* = 75)	Enzalutamide + LHRH vs. Enzalutamide + Abiraterone/Prednisolone + LHRH for 6 months	pCR 30% vs. 16% (*p* = 0.26)
McKay et al. [24]	Randomised Phase II	High-risk localised PCa (*n* = 118)	Apalutamide + Abiraterone/Prednisolone + LHRH vs. Abiraterone/Prednisolone + LHRH	pCR 22% vs. 20% 3-year BCRFS 81% vs. 72% (HR 0.81, 95% CI: 0.43–1.49)
ENZARAD phase III trial (ANZUP 1303/NCT0244644) [14]	Randomised Phase III	High-risk localised or locally advanced PCa (*n* = 802)	Enzalutamide 160 mg OD vs. non-steroidal anti-androgen for 6 months + 24 months of ADT and RT	Improved effects of Enzalutamide on MFS in two groups: N1 disease (*p* = 0.04) and planned pelvic RT (*p* < 0.001)
ATLAS trial (NCT0253516) [15]	Randomised Phase III	High-rish localised or locally advanced PCa (*n* = 1503)	Apalutamide + ADT vs. Placebo + ADT	Ongoing
Chemohormonal				
ACDC-RP (Fleshner et al.) [25]	Randomised Phase II	High-risk localised PCa (*n* = 70)	Abirateroner + LHRH ± Cabazitaxel for 6 months pre-RP	CR or MRD 44% Adding Cabazitaxel no added benefit
Alliance CALGB 90203 (update from 2020) [26]	Randomised Phase III	High-risk localised PCa (*n* = 788)	6 cycles Docetaxel + ADT vs. RP alone	Improved MFS (HR 0.70, 95% CI: 0.51–0.95) and OS (0.6, 95% CI: 0.40–0.94)
PEACE 2 [27]	Randomised Phase III	High-risk localised PCa	4 arms: (1) Prostate-only RT + 3 years ADT; (2) Prostate + Pelvic RT + 3 years ADT; (3) Prostate only RT + 3 years ADT + Cabzaitaxel; (4) Prostate + Pelvic RT + 3 years ADT + Cabzatiaxel	Results pending
DDR/PARP-Directed therapy				
NePtune (NCT05498272) [28]	Single-arm Phase II	High-risk/locally advanced BRCA 1/2^+^ PCa	Olaparib + LHRH for 6 months pre-RP	Ongoing Tolerability acceptance
GUNS (NCT04812366) [29]	Adaptive umbrella Phase II	High-risk PCa stratified by genomic alterations	LHRH + Apalutamide lead-in → Biomarker-matched arms	Ongoing Results indicating *ETS* fusion, *FOXA1*, *SPOP* associated with higher rates of MRD
Radioligand therapy				
LuTectomy [16]	Single-arm Phase I/II	High-risk PCA (ISUP 3–5, PSA > 20, cT2^+^, N1, PSMA SUV_max_ > 20)	One 5 GBq dose of [^177^Lu]Lu-PSMA-617 6 weeks pre-RP	45% had >50% PSA decline Well tolerated No surgical morbidity
Golan et al. [30]	Phase I	High-risk localised PCa	2–3 cycles of [^177^Lu]Lu-PSMA-617 pre-RP	Median PSA decline 34% after 3 cycles
PRELUDE [31]	Single-arm Phase II	High-risk localised PCa	[^177^Lu]Lu-PSMA-617 prior to RP	Ongoing aiming to define optimal dosing
Immunotherapy				
Shenderov et al. [32]	Single-arm Phase II	Intermediate/high-risk localised PCa (*n* = 32)	Enoblituzumab 6 weeks pre-RP	66% achieved undetectable PSA at 1 year Increased CD8^+^ T cell infiltration

## Data Availability

No new data was generated.

## Abbreviations

The following abbreviations are used in this manuscript:

ADT	Androgen deprivation therapy
AR	Androgen receptor
ARPI	Androgen receptor pathway inhibitor
AUA	American Urological Association
BCR	Biochemical recurrence
bRFS	Biochemical recurrence-free survival
CI	Confidence Interval
CRPC	Castration-resistant prostate cancer
DDR	DNA damage repair
EBRT	External beam radiotherapy
EAU	European Association of Urology
HRR	Homologous recombination repair
MFS	Metastasis-free survival
MRD	Minimal residual disease
NCCN	National Comprehensive Cancer Network
OS	Overall Survival
PCa	Prostate cancer
PARP	Poly (ADP-ribose) polymerase
PD-L1	Programmed death-ligand 1
PET	Positron emission tomography
pCR	Pathological complete response
PI3K	Phosphoinositide 3-kinase
PSA	Prostate-specific antigen
PSMA	Prostate-specific membrane antigen
RLT	Radioligand therapy
RP	Raidcal prostatectomy
RT	Radiotherapy
SUV	Standardised uptake value
TME	Tumour microenvironment
